# Immunomodulatory Effects of Polysaccharide from Marine Fungus *Phoma herbarum* YS4108 on T Cells and Dendritic Cells

**DOI:** 10.1155/2014/738631

**Published:** 2014-11-30

**Authors:** Song Chen, Ran Ding, Yan Zhou, Xian Zhang, Rui Zhu, Xiang-Dong Gao

**Affiliations:** School of Life Science and Technology, China Pharmaceutical University, 24 Tongjiaxiang, Nanjing 210009, China

## Abstract

YCP, as a kind of natural polysaccharides from the mycelium of marine filamentous fungus *Phoma herbarum* YS4108, has great antitumor potential *via* enhancement of host immune response, but little is known about the molecular mechanisms. In the present study, we mainly focused on the effects and mechanisms of YCP on the specific immunity mediated by dendritic cells (DCs) and T cells. T cell /DC activation-related factors including interferon- (IFN-) *γ*, interleukin-12 (IL-12), and IL-4 were examined with ELISA. Receptor knock-out mice and fluorescence-activated cell sorting are used to analyze the YCP-binding receptor of T cells and DCs. RT-PCR is utilized to measure MAGE-A3 for analyzing the tumor-specific killing effect. In our study, we demonstrated YCP can provide the second signal for T cell activation, proliferation, and IFN-*γ* production through binding to toll-like receptor- (TLR-) 2 and TLR-4. YCP could effectively promote IL-12 secretion and expression of markers (CD80, CD86, and MHC II) *via* TLR-4 on DCs. Antigen-specific immunity against mouse melanoma cells was strengthened through the activation of T cells and the enhancement of capacity of DCs by YCP. The data supported that YCP can exhibit specific immunomodulatory capacity mediated by T cells and DCs.

## 1. Introduction

T cells combine strict target specificity and high efficiency for tumor therapy [1]. Antigen-presenting cells (APCs) activate T cells through a two-signal mechanism: one is initiated by T cell receptor (TCR) binding to antigenic peptide presented by major histocompatibility complex (MHC) molecules and the second signal involves costimulatory molecules that interact with costimulatory receptors on the T cell surface and leads to T cell cytokine production and their proliferation [2]. Dendritic cells (DCs) are believed to be the most potent APCs which have the unique capacity to deliver antigens to T cells and express several costimulatory molecules [3].

The second signal required for T cell activation which supports cell survival, memory development, proliferation, and cytokines production found on the surface of DCs has been reported such as B7 family members B7-1 (CD80) and B7-2 (CD86) [4, 5]. Binding B7-1/B7-2 to CD28 is the strongest costimulatory signal delivered by DCs to provide a full activation of T cells, promoting their proliferation and IL-2 secretion [6, 7]. CD80 and CD86 have been reported to have particular functions in eliciting T cell activation and inducing differential patterns of cytokine expression supporting type 1 or type 2 T-helper (Th1 or Th2) response upon binding to CD28 [2, 8]. The primary outcome of CD28-mediated stimulation on molecular level is an increased production of cytokines such as IL-2 which is important for T cell proliferation, antiapoptosis [6].

Toll-like receptors (TLRs), as a family of pattern-recognition receptors (PRRs), are highly expressed on DC and T cell [9]. Activation of TLR leads to DC maturation and secretion of proinflammatory cytokines, which can induce T cell antitumor immune response [10]. Many polysaccharides as TLR agonists that function as adjuvant and stimulate DCs to prime antigen-specific T and B cell responses have been reported [11–13]. On T cells, pretreatment with TLR4 ligand LPS enhanced their survival and increased their suppressive activity, whereas TLR4 deficient mice did not respond [14]. Both TLR and TCR signaling pathways utilize members of the MAPK family. TLR activation of these pathways influences the subsequent TCR-mediated signaling events [15, 16]. TLR agonists can induce activation of CD4^+^ T lymphocytes, CD8^+^ T lymphocytes, or cytotoxic T lymphocytes (CTLs) [17–19]. These findings prompt that TLR agonists may cause the activation of DC and provide signal required for T cell activation.

YCP (YCP is the acronym of Yancheng polysaccharide) was purified from the mycelium of* Phoma herbarum* YS4108 that inhabits the sediment in the Yellow Sea area around Yancheng, China. It has a backbone of *α*-1,4-D-glucan with a lower proportion of *α*-1,6-linked glucopyranosyl and glucuronic acid residues as nonreducing terminals, and we have previously found that it possesses a great antitumor potential* via* enhancement of host immune response [20, 21]. However, further studies are still needed to clarify the molecular mechanism of YCP action. In this study, we mainly focus on the effects and mechanisms of YCP on the specific immunity mediated by DCs and T cells.

## 2. Materials and Methods

### 2.1. Materials

YCP was isolated and characterized in our lab previously [21]. All primary antibodies were purchased from eBioscience (San Diego, CA, USA) and used at concentrations between 1 and 5 *μ*g/mL. 1-Cyano-4-dimethylaminopyridinium tetrafluoroborate (CDAP) was purchased from Sigma Chemical Co. (St. Louis, MO, USA). Recombinant murine IL-4 and recombinant murine GM-CSF were purchased from PeproTech (Rocky Hill, NJ, USA). ELISA kits for measuring murine IFN-*γ*, IL-4, and IL-12 were purchased from R&D Systems (Minneapolis, MN, USA). Anti-mouse TLR2 (CD282) and anti-mouse TLR4 were purchased from eBioscience (San Diego, CA, USA). Pam3CSK4 was purchased from Santa Cruz (San Cruz, CA, USA). 3-(4, 5-Dimethylthiazol-2-yl)-2, 5-diphenyltetrazolium bromide (MTT), ophenylenediamine, and fluoresceinamine (FLA) were from Sigma-Aldrich (St. Louis, MO, USA). RNA simple Total RNA Kit was purchased from Tiangen Biotech (Beijing, China). First-Strand cDNA Synthesis Kit was purchased from TransGen Biotech (Beijing, China). SYBR Green was purchased from Applied Biosystems (Foster City, CA, USA). Reagents used for cell culture were purchased from Gibco (Grand Island, NY, USA). All other reagents used in this study were of the highest available quality.

### 2.2. Experimental Animals

Male C57BL/6 mice of clean grade (6–8 weeks old) weighing 18–22 g were purchased from the Comparative Medicine Centre of Yangzhou University and acclimatized for 1 week prior to use. B6.129-Tlr2^tm1Kir^/JNju (TLR2 KO) and C57BL/10ScNJNju (TLR4 KO) mice between 6 and 8 weeks old were provided by the Model Animal Research Center of Nanjing University (Nanjing, Jiangsu, China). Throughout the study, the animals were fed by rodent laboratory chow pellets and tap water and were kept with five in each plastic cage in a well-ventilated room, maintained at 21 ± 2°C with humidity of 50 ± 10% and a 12/12 h light/dark cycle. All the procedures were carried out in strict accordance with the China legislation on the use and care of laboratory animals and were approved by the university committee for animal experiments.

### 2.3. Isolation of T Cells

Mouse splenic T cells were prepared according to the method described previously [22] with slight modification. Briefly, murine spleens were harvested from C57BL/6 inbred mice and minced in RPMI-1640 medium at room temperature. Erythrocytes were removed by hypotonic lysis using NH_4_Cl and adherent cells were separated by plating at 37°C for 6 h. The suspended cell populations were collected and used as the lymphocyte populations and applied to a nylon fiber column (Wako, Osaka, Japan). After the cell suspension sank into the fiber bed thoroughly, the columns were incubated at 37°C for 1 h. T cells were gently eluted without adding any additional pressures and B cells were flushed out of the column using a plunger. The purity of T cells was characterized through fluorescence-activated cell sorting (FACS) analysis for CD3 expression; the ratio of CD3^+^ T cells was approximately 90%.

### 2.4. Binding and Competition Assay

YCP was conjugated with FLA by using the CDAP-activation method as previously described with slight modification [23]. Briefly, 5 mg CDAP was added into an aqueous solution containing 30 mg of YCP with gentle stirring and maintained at pH 9.0 for 2.5 min. The CDAP-activated YCP was then mixed with 2 mg of FLA (pH adjusted to 8.0) and incubated at room temperature overnight. Fluoresceinamine-labeled YCP (fl-YCP) was separated from the excess-free FLA with an Amicon Ultra-15 centrifugal filter unit (Millipore, Billerica, MA, USA). The FLA and YCP amounts in fl-YCP were, respectively, quantified by measuring absorbance at 440 nm and phenol-sulfuric acid assay [20]. To perform the competition assay, T cells were incubated with medium, Pam3CSK4 (20 nM), LPS (20 nM), Pam3CSK4 (20 nM) + LPS (20 nM), and anti-mouse CD28 (5 *μ*g/mL) for 1 h, followed by incubation with fl-YCP (100–1600 nM) for 1 h. After washing three times with PBS, cells were examined on a FACSCalibur flow cytometer (BD Biosciences, San Jose, CA, USA) with a 488 nm laser excitation and a 530 nm emission filter. Data were acquired from a minimum of 100 000 cells and analyzed using the FlowJo program (FreeStar, Ashland, OR, USA).

### 2.5. Proliferation Assay

T cells were cultured in 96-well microplates at a density of 2 × 10^6^ cells/mL in RPMI-1640 medium containing 10% FBS, supplemented with 60 mg/L penicillin and 100 mg/L streptomycin. The cells were pretreated with medium or anti-mouse CD3 (5 *μ*g/mL) at 4°C for overnight culture and stimulated with YCP (100–800 nM) for 48 h in a CO_2_ incubator or with anti-TLR2 (20 nM), anti-TLR4 (20 nM), anti-TLR2 (20 nM) + anti-TLR4 (20 nM), and medium at 37°C for 2 h prior to addition of YCP (400 nM), followed by MTT (5 mg/mL) for another 4 h. The formazan crystals formed from MTT by living cells were fully dissolved in DMSO for 10 min. The absorbance was determined at 570 nm in a multiskan spectrum (Thermo Fisher Scientific, Vantaa, Finland), and induction of cell proliferation was expressed as the proliferation index, calculated by dividing* A*570 of stimulated cells with* A*570 of control cells [22].

### 2.6. Generation and Maturation of Bone Marrow-Derived DCs

Bone marrow was derived from 6–8-week-old male C57BL/6 mice. Bone marrow-derived dendritic cells (BMDCs) were generated by culturing monocytes for 7 days in RPMI 1640 medium, 10% FBS, 1% penicillin/streptomycin, 2 mM L-glutamine, 100 ng/mL recombinant murine granulocyte-macrophage colony stimulating factor (GM-CSF) (PeproTech), and 50 ng/mL recombinant murine IL-4 (PeproTech) [24]. Every 2 days, 50% of the culture media was exchanged with fresh media. DCs were washed by PBS and stained with anti-mouse CD11c FITC monoclonal antibody (eBioscience, San Diego, CA, USA).

An established stable mouse melanoma cell line, B16F10, was used (kindly donated by Professor Heng Zheng). Cells were cultured in RPMI-1640 medium containing 10% FBS, supplemented with 60 mg/L penicillin and 100 mg/L streptomycin. B16F10 cells were resuspended at a density of 1 × 10^7^ cells/mL in a 1.5 mL Eppendorf tube with water bath for 30 min at 40°C and then putted into −80°C refrigerator for 20 min, slow thawing at 37°C for 20 min. After 4 freeze-thaw cycles, cells were centrifuged at 3000 rpm for 20 min. The supernatant was passed through a 22 *μ*m sieve called B16F10 full-cell antigen (B16Ag). Adjust the concentration of B16Ag to 0.1 mg/mL. DCs cultured for 7 days were stimulated by B16Ag for 48 h. DCs (mDCs or imDCs) were washed by PBS and stained with anti-mouse MHC class II PE and anti-mouse CD11c FITC monoclonal antibodies (eBioscience, San Diego, CA, USA). For another experiment, DCs cultured for 7 days as described in Section 2.7 were stimulated by B16Ag and YCP (100–800 nM) for 48 h. DCs were washed by PBS at various times (6 h, 12 h, 24 h, and 48 h) and stained with anti-mouse CD80 (B7-1) FITC and anti-mouse CD86 (B7-2) PE monoclonal antibodies (eBioscience, San Diego, CA, USA). Data were acquired from a minimum of 20 000 cells and analyzed using the FlowJo program.

### 2.7. ELISA

T cells were cultured in 96-well microplates at a density of 2 × 10^6^ cells/mL pretreated with medium or anti-mouse CD3 (5 *μ*g/mL) at 4°C for overnight culture and stimulated with YCP (100–800 nM) for 48 h in a CO_2_ incubator or with anti-TLR2 (20 nM), anti-TLR4 (20 nM), anti-TLR2 (20 nM) + anti-TLR4 (20 nM), and medium at 37°C for 2 h prior to addition of YCP (400 nM). Cell-free supernatants were collected for quantification of IFN-*γ* level by commercial ELISA kits according to the manufacturer's protocol described previously [20].

B16F10 peptide-pulsed DCs were cultured in 96-well microplates at a density of 2 × 10^6^ cells/mL in RPMI-1640 medium containing 10% FBS, supplemented with 60 mg/L penicillin and 100 mg/L streptomycin. B16Ag-DCs (mDCs) were stimulated with YCP (100–800 nM) for 48 h in a CO_2_ incubator or with anti-TLR2 (20 nM), anti-TLR4 (20 nM), anti-TLR2 (20 nM) + anti-TLR4 (20 nM), and medium at 37°C for 2 h prior to addition of YCP (400 nM). Cell-free supernatants were collected for quantification of IL-12 level by commercial ELISA kits according to the manufacturer's protocol described previously [20].

### 2.8. *In Vitro* Activation of T Cells and Induction of Antigen-Specific Responses by mDCs

T cells were cultured with mDCs at the ratio of 20 : 1 or without mDCs for 48 h as effector cells (2 × 10^6^ cells/mL). The B16F10 cells were resuspended at a density of 2 × 10^5^ cells/mL as target cells. The effector cells and target cells were cocultured and stimulated by medium or YCP (100–800 nM) for 48 h. Cells were collected for real-time quantity RT-PCR. The supernatants were obtained and the levels of IFN-*γ*, IL-12, and IL-4 productions were measured by ELISA (R&D Systems) according to the manufacturer's protocol as described in Section 2.7. Cells stimulated by medium and cells stimulated by YCP (400 nM) were collected at various times (0 h, 6 h, 12 h, 24 h, 48 h, and 96 h) after washing by PBS and stained with anti-mouse CD3e PE-Cyanine5, anti-mouse CD4 FITC, and anti-mouse CD8a PE monoclonal antibodies (eBioscience, San Diego, CA, USA).

### 2.9. Quantitative Real-Time RT-PCR

The number of target cells was measured by the mRNA quantitation of MAGE-A3. Total RNA was isolated from cell pellet samples using the RNA simple Total RNA Kit from Tiangen Biotech (Beijing, China). RNA recovered (about 2 *μ*g) was reverse transcribed to cDNA using random hexamer primers and First-Strand cDNA Synthesis Kit from TransGen Biotech (Beijing, China). B16F10 cells proliferation was assessed by measuring MAGE-A3 gene expression using the ABI Prism 7500 Sequence Detection System to perform qRT-PCR as already described in detail [25]. Briefly, PCR was conducted in 10 *μ*L using 20% of the recovered cDNA as the template, with 2 *μ*M GAPDH (Internal Standard) primers (forward, 5′-AGAAGGCTGGGGCTCATTTG-3′, 1.5 *μ*L; reverse, 5′-AGGGGCCATCCACAGTCTTC-3′, 1.5 *μ*L) or 1 *μ*M MAGE-A3 specific primers (forward, 5′-AGCTCTGCATCGTTTTGGGTT-3′, 1.5 *μ*L; reverse, 5′-GTTCCATTATCCGCTACATCTGAA-3′, 1.5 *μ*L) and 5 *μ*L 2× SYBR Green from Applied Biosystems (Foster City, CA, USA). Cycling parameters were 95°C 20 s, followed by 40 cycles of 95°C 20 s and 60°C 30 s. Melt curve parameters were 95°C 15 s, 60°C 60 s, and 95°C 15 s.

### 2.10. *In Vitro* Cell Models to Study the YCP-Mediated Specific Immunity against Mouse Melanoma Cells

Four cell models were prepared to study the signal provided by YCP during* in vitro* specific immune responses. The matured DCs (WT and TLR4 KO) and T cells (WT and TLR4 KO) were cocultured according to the ratio of 1 : 10 while DCs were resuspended at a density of 2 × 10^5^ cells/mL for 48 h. The mixed cells after being cocultured were used as effector cells (2.2 × 10^6^ cells/mL), and the B16F10 cells resuspended at a density of 2.2 × 10^5^ cells/mL were used as target cells. The effector cells and target cells were cocultured and stimulated by medium or YCP (400 nM) for 48 h. The number of target cells was measured by quantitative real-time RT-PCR (qRT-PCR) as described in Section 2.9. The supernatants were obtained and the levels of IFN-*γ*, IL-12, and IL-4 productions were measured by ELISA (R&D Systems) according to the manufacturer's protocol as described in Section 2.8.

### 2.11. Statistical Analysis

The Prism 5.0 program (GraphPad Software, La Jolla, CA, USA) was used for statistical analysis. All results were expressed as mean ± standard deviation (SD). Each value is the mean of at least three separate experiments. Mann-Whitney *U* test or Kruskal-Wallis test followed by Dunn's post hoc test was performed to determine significant differences where appropriate.

## 3. Results

### 3.1. YCP Acts as the Second Signal in TCR Activation on T Cell

**Figure 1:**
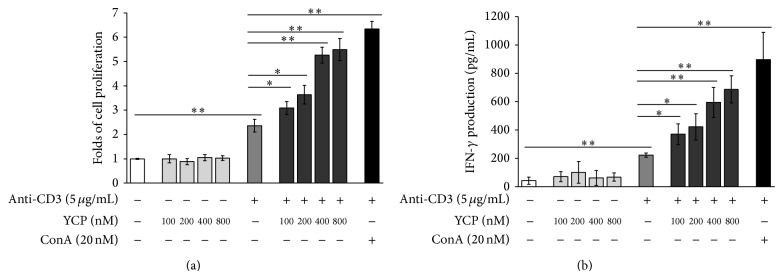
The effects of YCP on T cell proliferation and IFN-*γ* production as the second signal in TCR activation. (a) Effect of YCP on T cell proliferation. Splenic T cells prestimulated with aCD3 or PBS were treated with YCP or ConA at the indicated concentrations, and cell proliferation was measured by MTT assay at 48 h (*n* = 6, ^*^ *P* ≤ 0.05, ^**^ *P* ≤ 0.01 by Kruskal-Wallis test followed by Dunn's post hoc test). (b) Effect of YCP on T cell IFN-*γ* production. Splenic T cells prestimulated with aCD3 or PBS were treated with YCP or ConA at the indicated concentrations, and IFN-*γ* production was measured by ELISA assay at 48 h (*n* = 6, ^*^ *P* ≤ 0.05, ^**^ *P* ≤ 0.01 by Kruskal-Wallis test followed by Dunn's post hoc test).

Splenic T cells were isolated by nylon fibers to a purity >90% (data not shown) and then cultured with YCP or ConA as the positive control in the 96-well plate which had been precoated with aCD3 functional antibody or PBS. T cell proliferation was measured after 48 h by MTT assay. The results indicated that YCP could not stimulate T cell alone. With the precoated aCD3, YCP could significantly stimulate T cell to proliferate in a dose-dependent manner (Figure 1(a)). In addition to the proliferative effect, the effect of YCP on cytokine production from T cells was evaluated. The supernatants collected from naïve and YCP-stimulated cells were tested by ELISA. With the precoated aCD3, YCP could significantly stimulate T cell to produce IFN-*γ* in a dose-dependent manner (Figure 1(b)).

The stimulation obtained with YCP on T cell suggests that YCP may be an efficacious second signal in TCR activation just like the contribution of costimulatory molecules on T cell stimulant capable of promoting cell proliferation and producing IFN-*γ*.

### 3.2. The Enhancement Effect of YCP on DCs

**Figure 2:**
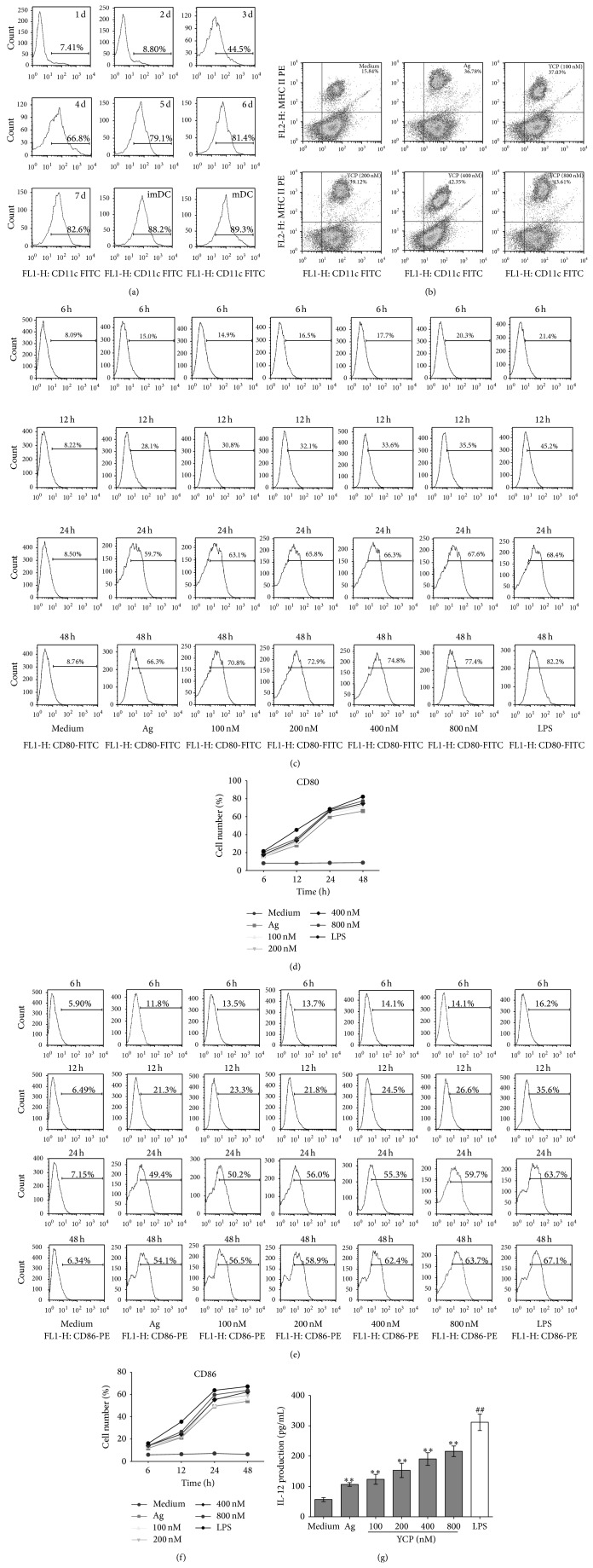
The effect of YCP on DCs. (a) The expression of CD11c on DCs after 7-day generation with GM-CSF and IL-4. (b) The percentage of mature DCs (CD11c^+^/MHC II^+^) stimulated by Ag and YCP at the indicated concentrations. ((c) and (d)) CD80 on dendritic cells at different times (6 h, 12 h, 24 h, and 48 h) after the stimulations with medium/Ag/YCP+Ag/LPS+Ag. ((e) and (f)) CD86 on dendritic cells at different times (6 h, 12 h, 24 h, and 48 h) after the stimulations with medium/Ag/YCP+Ag/LPS+Ag. (g) Effect of YCP on DC IL-12 production. DCs were treated with Ag or YCP+Ag or LPS at the indicated concentrations, and IL-12 production was measured by ELISA assay at 48 h (*n* = 6, ^*^ *P* ≤ 0.05, ^**^ *P* ≤ 0.01 versus medium by Kruskal-Wallis test followed by Dunn's post hoc test. ^##^ *P* ≤ 0.01 versus medium by Mann-Whitney *U* test).

DCs generated with GM-CSF and IL-4 as immature DCs expressed amounts of CD80, CD86, and MHC class II molecules as fully matured DCs. To determine the purity of monocyte-derived dendritic cells, CD11c was detected by flow cytometry every day. After 7 days generation with GM-CSF and IL-4, the expression of CD11c has been significantly increased to 82.6%. And then followed by medium or B16F10Ag for 48 h, the expression of CD11c was 88.2% and 89.3%, respectively (Figure 2(a)). To determine the maturation of DCs, the pattern of expression of various cell-surface makers encoding MHC II, CD80 (B7-1), and CD86 (B7-2) was detected by flow cytometry. There were more matured DCs that coexpressed both MHC class II and CD11c stimulated by Ag and YCP (Figure 2(b)). CD80 and CD86 on DCs at different times (6 h, 12 h, 24 h, and 48 h) after the stimulations with medium/Ag/YCP+Ag/LPS+Ag were analyzed by using the FlowJo program (Figure 2(c)–2(f)). With the stimulation of B16F10Ag, the expressions of CD80 and CD86 on DCs significantly increased to 66.3% and 54.1% at 48 h by contrast with medium group. Such stimulation was delayed up to 24 h after maturation. YCP assisted the augments of the expressions of CD80 and CD86 on DCs suggesting that YCP could promote the maturation of DCs.

DCs cultured for 7 days were stimulated with medium/Ag/YCP+Ag/LPS+Ag for 48 h. The supernatants were obtained and the levels of IL-12 productions were measured by ELISA. YCP could significantly stimulate DCs to produce IL-12 in a dose-dependent manner (Figure 2(g)). The stimulation mediated by YCP on DCs suggested that YCP is an activator of cell-surface molecule (MHC class II, CD80, and CD86) expression and IL-12 production on DCs.

### 3.3. Toll-Like Receptor Is the Receptor for YCP on T Cells and DCs

**Figure 3:**
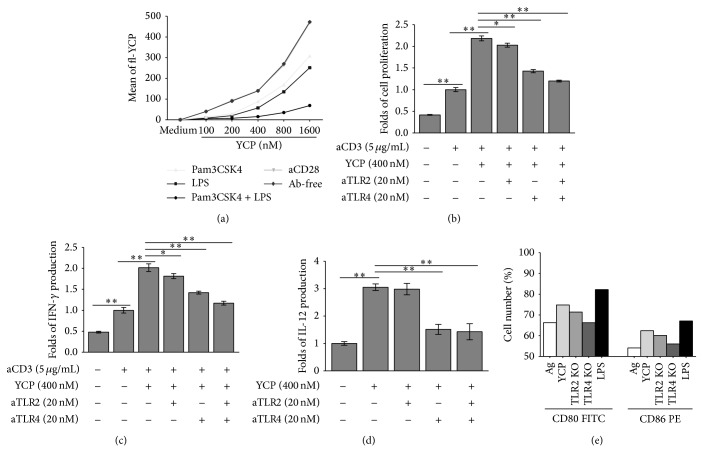
The roles of TLR2 and TLR4 in the actions of YCP on T cells and DCs. (a) Ligand competition experiment on T cells. Splenic T cells were incubated with Pam3CSK4 (a bona fide TLR2 ligand), LPS (a bona fide TLR4 ligand), and anti-mouse CD28 (CD28 ligand) for 1 h, followed by fl-YCP (100–1600 nM) for 1 h in competition experiment analyzed by flow cytometry. ((b) and (c)) Splenic T cells prestimulated with aCD3 or PBS were pretreated with anti-TLR2, anti-TLR4, or both (20 nM) for 2 h before the addition of YCP. After 48 h incubation, cell proliferation (b) and IFN-*γ* concentration in supernatants (c) were measured using MTT assay or ELISA, respectively (*n* = 6, ^*^ *P* ≤ 0.05, ^**^ *P* ≤ 0.01 by Mann-Whitney *U* test). (d) DCs were pretreated with anti-TLR2, anti-TLR4, or both (20 nM) for 2 h before the addition of YCP. After 48 h incubation, IL-12 concentration in supernatants was measured by ELISA assay (*n* = 6, ^*^ *P* ≤ 0.05, ^**^ *P* ≤ 0.01 by Mann-Whitney *U* test). (e) The expressions of CD80 and CD86 treated by Ag (WT)/YCP+Ag (WT, TLR2 KO mice, TLR4 KO mice)/LPS (WT) were evaluated on DCs.

TLRs played a direct role in control of T cell activation including proliferation, cytokine secretion, upregulation of activation markers, and terminal differentiation. As the receptors on B cells of YCP, TLR2, and TLR4 had been reported in our previous study, based on these findings, we focused our study on the functional relevance of TLR2 and TLR4 in YCP-mediated TCR activation. Fluoresceinamine-labeled YCP (fl-YCP) (100–1600 nM) was prepared. T cells were incubated with Pam3CSK4 (a bona fide TLR2 ligand), LPS (a bona fide TLR4 ligand), and anti-mouse CD28 (CD28 ligand) for 1 h, followed by fl-YCP (100–1600 nM) for 1 h in competition experiment analyzed by flow cytometry (Figure 3(a)). The results suggested that YCP as the ligand of TLR2 and TLR4 could compete with Pam3CSK4 and LPS. CD28 was not the binding target of YCP; in other words, the YCP T cell activation was achieved by signal provided by TLR without the signal of B7-1/B7-2:CD28 pathway. To prove this conclusion, we blocked receptors with specific antibodies. T cells were stimulated with YCP in the presence of anti-TLR2 or anti-TLR4 and then subjected to proliferation assay and total IFN-*γ* quantification. Antibodies to TLR2 and TLR4 significantly reduced the proliferation effects of YCP and suppressed the induction of IFN-*γ* production with the presence of aCD3 (functional antibody) which provided first signal for activation of TCR (Figures 3(b) and 3(c)). With the presence of anti-TLR2, T cell proliferation was reduced by 12.1%. With the presence of anti-TLR4, T cell proliferation was reduced by 39.3%. With the presence of anti-TLR2, the production of IFN-*γ* was decreased by 13.1%. With the presence of anti-TLR4, the production of IFN-*γ* was decreased by 33.8%.

The reduction was found to be synergetic upon the combined treatment with anti-TLR2 and anti-TLR4. Therefore, we proposed that TLR2 and TLR4 might be functionally correlated with activation of TCR by YCP as second signal.

As the main receptors of DCs, TLRs have been reported to be important binding targets of polysaccharide vaccine. Anti-TLR4 significantly suppressed the induction of IL-12 production (51.8%). To further confirm this statement, the impact that loss of TLR2 or TLR4 has upon YCP activities was evaluated in DCs from either B6.129-Tlr2^tm1Kir^/JNju (TLR2 KO) or C57BL/10ScNJNju (TLR4 KO) mice. The upregulations of CD80 and CD86 were reduced on TLR2 KO (2.5% and 1.4%) and TLR4 KO mice (8.5% and 5.6%), especially on TLR4 KO mice (Figure 3(e)). These findings provided convincing evidence that TLR4 was responsible for activation on DCs by YCP.

### 3.4. YCP Strengthens Specific Immunity against Mouse Melanoma Cells through Activation of T Cells and Enhancement of Capacity of DCs

**Figure 4:**
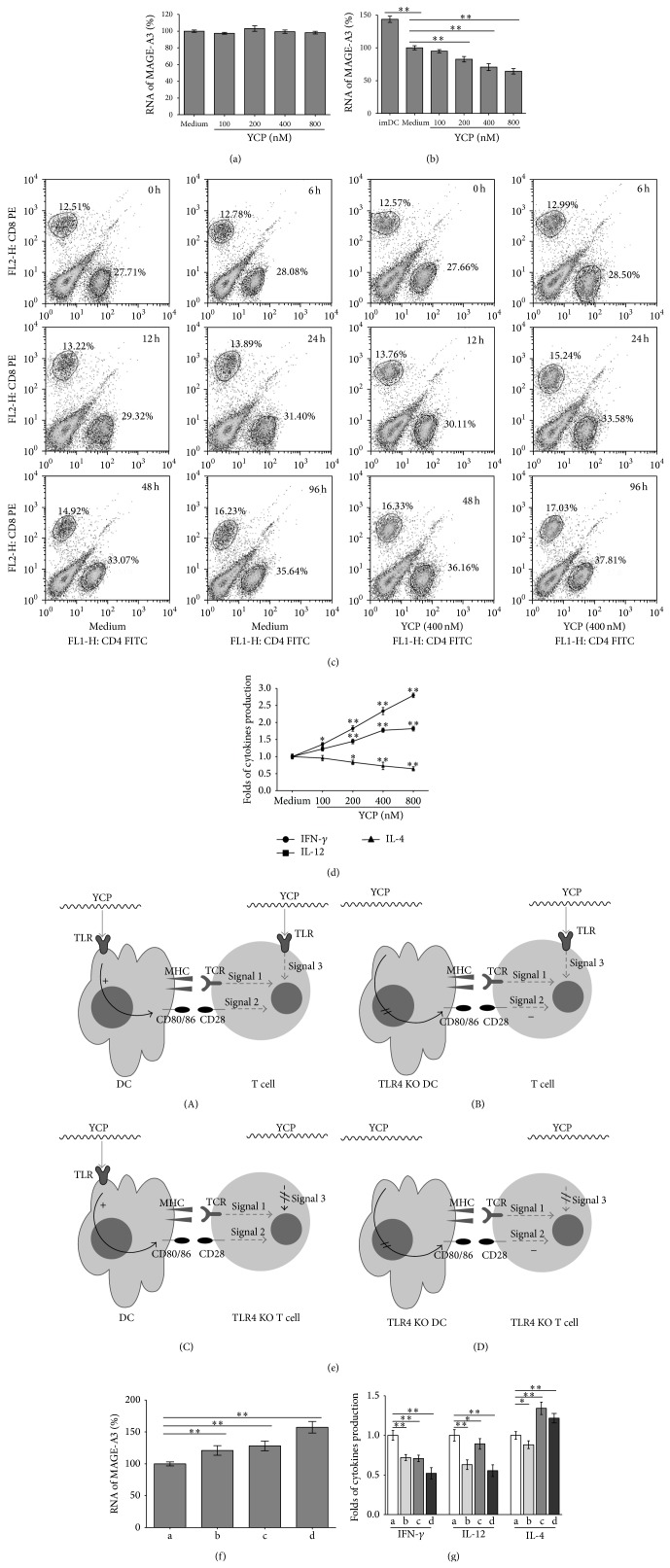
The effect and mechanism of YCP on the specific immunity enhancement. (a) The target cells (B16F10 cells) stimulated by medium or YCP (100–800 nM) for 48 h directly. The number of target cells was measured by the mRNA quantitation of MAGE-A3. (b) The effector cells (T cells were cultured with mDCs at the ratio of 20 : 1 for 48 h) and target cells (B16F10 cells) were cocultured and stimulated by medium or YCP (100–800 nM) for 48 h. The number of target cells was measured by the mRNA quantitation of MAGE-A3. (c) The populations of CD4^+^ T cells and CD8^+^ T cells treated by medium or YCP (400 nM) were detected by flow cytometry during the incubation (0 h, 6 h, 12 h, 24 h, 48 h, and 96 h). (d) The expressions of IFN-*γ*, IL-12, and IL-4 concentration in supernatants were measured using ELISA assay after the incubation at 48 h. (e) Four cell groups were prepared to study the signal provided by YCP during* in vitro* specific immune responses as follows: (a) matured DCs (WT) and T cells (WT), (b) matured DCs (TLR4 KO) and T cells (WT), (c) matured DCs (WT) and T cells (TLR4 KO), and (d) matured DCs (TLR4 KO) and T cells (TLR4 KO). (f) The effector cells (T cells were cultured with mDCs at the ratio of 20 : 1 for 48 h) and target cells (B16F10 cells) were cocultured and stimulated by medium or YCP (100–800 nM) for 48 h. The number of target cells in each group was measured by the mRNA quantitation of MAGE-A3. (g) The expressions of IFN-*γ*, IL-12, and IL-4 concentration in supernatants were measured using ELISA assay after the incubation in each group at 48 h (*n* = 6, ^*^ *P* ≤ 0.05, ^**^ *P* ≤ 0.01 by Mann-Whitney *U* test).

DCs cultured for 7 days were stimulated by B16Ag for 48 h. T cells were cultured with mDCs at the ratio of 10 : 1 for 48 h as effector cells (about 2 × 10^6^ cells/mL). The B16F10 cells were resuspended at a density of 2 × 10^5^ cells/mL as target cells. The effector cells and target cells were cocultured and stimulated by medium or YCP (100–800 nM) for 48 h. The number of target cells was measured by the mRNA quantitation of MAGE-A3. YCP could not kill target cells directly (without the effector cells) (Figure 4(a)) and could significantly decrease the mRNA of MAGE-A3 on target cells with the incubation of DCs and T cells in a dose-dependent manner (Figure 4(b)). The results indicate that YCP could strengthen the destruction of target cells in a dose-dependent manner.

During the incubation, the populations of CD4^+^ T cells and CD8^+^ T cells have been increased significantly in 96 h (Figure 4(c)). The presence of YCP could strengthen this phenomenon that both CD4^+^ T cells and CD8^+^ T cells have been improved (Figure 4(c)). The ratio of CD4^+^/CD8^+^ is kept stable at 2.20 ± 0.01 (medium) to 2.22 ± 0.02 (YCP) which means T cells maintain the biological homeostasis. The levels of IFN-*γ*, IL-12, and IL-4 productions were measured by ELISA. The production of IFN-*γ* and IL-12 reached higher levels and the production of IL-4 was suppressed to a lower level (Figure 4(d)). The results indicate that YCP enhances the cytotoxicity and cytokine production and promotes activation of APCs and the cell-mediated immunity.

### 3.5. The Mechanism of the Specific Immunity Enhancement Mediated by YCP

As TLR4 was the receptor of YCP on DC and T cell, four cell groups were prepared to study the signal provided by YCP during* in vitro* specific immune responses as follows (Figure 4(e)): (a) matured DCs (WT) and T cells (WT), (b) matured DCs (TLR4 KO) and T cells (WT), (c) matured DCs (WT) and T cells (TLR4 KO), and (d) matured DCs (TLR4 KO) and T cells (TLR4 KO).

DCs from different groups cultured for 7 days were stimulated by B16Ag for 48 h. T cells from different groups were cultured with mDCs at the ratio of 10 : 1 for 48 h as effector cells (2.2 × 10^6^ cells/mL). The B16F10 cells were resuspended at a density of 2 × 10^5^ cells/mL as target cells. The effector cells and target cells were cocultured and stimulated by medium or YCP (400 nM) for 48 h. The mRNA level of MAGE-A3 on target cells (Figure 4(f)) and the levels of IFN-*γ*, IL-12, and IL-4 productions were measured (Figure 4(g)). The results indicated that YCP can suppress the production of IL-4 and induce the production of IL-12 and IFN-*γ* *via* TLR4 on T cells, while inducing the production of IL-4, IL-12, and IFN-*γ* *via* TLR4 on DCs from these* in vitro* cell models for studying the YCP-mediated specific immunity. The target cell killing was enhanced with the activations on T cells and DCs* via* TLR4 induced by YCP.

## 4. Discussion

Many classes of biomacromolecules containing carbohydrate structures have been shown to exert a successful immune response. In our previous study YCP was demonstrated to stimulate the activation of macrophages and B cells [20, 26], and in recent years some polysaccharides that function as adjuvant and stimulate DC to induce T cell responses have been reported [11–13]. However, little is known about the molecular mechanisms that how YCP could interfere with the specific immunity mediated by DC and T cell. Thus, we focus on how YCP performs its function on DCs and T cells. Our current study indicates that YCP-induced TLR activation can induce TCR activation at the presence of functional antibody aCD3 [27] which is the “first” signal of TCR activation on T cells. When the “first” signal is provided from DC, YCP could also enhance the TCR activation.

LPS is used in other studies to enhance antigen-driven proliferation on DC [28, 29]. The functional maturation of dendritic cells has been shown previously to be associated with increased levels of certain T cell costimulatory molecules [24, 30, 31]. Bone marrow-derived dendritic cells generated in the presence of GM-CSF and IL-4 exhibit an intermediate maturation stage with respect to phenotype and* in vitro* antigen-presenting capacity [24]. In our study, treatment with LPS can result in increasing levels of markers on DCs which is in agreement with previous observations. Furthermore, LPS will modify B7 (B7-1 and B7-2) expression of monocytes [31]. YCP is demonstrated which could increase the expression of CD80, CD86, and MHC-II in DCs and then strongly enhances T cell activation. Then, activated T cells can produce IFN-*γ*. Furthermore, the secretion of Th1-type cytokines IL-12 and IFN-*γ* was increased while Th2-type cytokine IL-4 was decreased by YCP* in vitro*. Induction of this immune response is a desirable characteristic for an immune adjuvant. The T-helper cell amplifies itself by secreting lymphokines. This action of YCP can enhance the helper cell response as well as the immune system's response to foreign antigens. Similar effects of other fungal carbohydrates have been reported by several existing literatures. Wang et al. [32] found that Sarcodon imbricatus polysaccharide (SIP) could prominently promote the production of IL-2 and IFN-*γ*, indicating that SIP could positively regulate the immunoreactions; meanwhile SIP significantly decreased the content of IL-4 which negatively regulated the immunoreactions. Yoshino et al. [33] investigated the effect of lentinan on modulating Th1 and Th2 responses in patients with digestive cancers. After lentinan treatment, CD4^+^ IFN-*γ* ^+^ T cell percentages increased significantly, whereas CD4^+^ IL-4^+^ T cell percentage decreased significantly, suggesting that lentinan may improve the balance between Th1 and Th2. A polysaccharide designated as the D-fraction, isolated from maitake (*Grifola frondosa*), can reduce the expression of Th2 cytokine IL-4 but markedly increase the expression of Th1 cytokine IFN-*γ* in CD4^+^ T cells and also increase IL-12p70 production, indicating that D-fraction promotes the differentiation into Th1 cells of CD4^+^ T cells [34]. CD4^+^ T cells augment the immune response by secreting cytokines that stimulate either a cytotoxic T cell response (Th1 cytokine) or an antibody response (Th2 cytokine) [35]. In addition to the results of the present study, our previous study showed that YCP can also generate a robust antibody response on B cells. Melanoma associated antigens (MAGEs) are found in melanoma as tumor-associated antigens (TAAs) and some of these proteins, such as MAGE-1, MAGE-A3, and MAGE-A10, are believed to be the detection and diagnostic target of melanoma [36]. MAGE-A3, which is not expressed in noncancer cells, is highly specific tumor mRNA marker, and it can be recognized by T cells which attack the B16F10 cells in a highly specific manner [37–39]. YCP could not kill melanoma cells directly but could significantly decrease the number of target cells with the incubation of DCs and T cells.

TLR agonists, such as Pam3CSK4, LPS, are demonstrated to promote DC and T cell activation and the engagement of TLRs in the induction of DC and T cell has been studied in the last few years [40, 41]. Competitive inhibition at TLR2 and TLR4 by using Pam3CSK4, LPS as ligands with fl-YCP, indicates the potential receptors of YCP on T cell. Antibody blocking experiment and gene defect experiment provide convincing evidence that TLR4 is responsible for activation on DC by YCP and TLR2 and TLR4 are responsible for T cell activation by YCP.

## 5. Conclusions

In conclusion, we demonstrate that YCP acts as the “second” signal of T cell activation to promote T cell proliferation and IFN-*γ* secretion* via* TLR2 and TLR4. YCP can effectively promote IL-12 secretion and expression of markers (CD80, CD86, and MHC II)* via* TLR4 on DCs. The data support that YCP is a good candidate for tumor-specific immunotherapy to enhance signals of antigen presentation process, antigen-specific T cell proliferation, Th1 cytokine induction, and tumor-specific killing.
